# Enigma of social media use: complexities of social media addiction through the serial mediating effects of emotions and self-presentation

**DOI:** 10.3389/fpsyg.2024.1448168

**Published:** 2024-12-06

**Authors:** Wing W. Y. Ho, Yan H. Y. Lau, Leona Y. L. Leung, Eric K. L. Li, Reyna K. K. Ma

**Affiliations:** ^1^School of Education and Languages, Hong Kong Metropolitan University, Kowloon, Hong Kong SAR, China; ^2^School of Nursing and Health Studies, Hong Kong Metropolitan University, Kowloon, Hong Kong SAR, China; ^3^Department of Sports Science and Physical Education, The Chinese University of Hong Kong, Shatin, Hong Kong SAR, China

**Keywords:** serial mediation, social media addiction, social media use, positive and negative affect, fear of missing out, offline and online self-presentation

## Abstract

**Introduction:**

Excessive social media use, though considered unhealthy, is no longer formally categorized as an addiction or disorder, leading to a lack of consensus on this behavior. It raises concerns regarding the exclusion of Internet Addiction Disorder from the DSM-5-TR due to insufficient empirical evidence. This study investigates the serial mediating effects of positive and negative affect, fear of missing out, and offline and online self-presentation in the relationship between social media use and social media addiction.

**Method:**

The study was conducted in Hong Kong with 385 participants (84.2% F, age range = 18–60) of an online survey. This study administered the Social Media Use Scale, Positive and Negative Affect Schedule, Fear of Missing Out Scale, Presentation of Online Self Scale, and Bergen Social Media Addiction Scale.

**Results:**

The results indicate that social media use plays a significant role in predicting both positive and negative affect. The findings further reveal that positive and negative affect, fear of missing out, and offline and online self-presentation act as serial mediators in the relationship between social media use and social media addiction. In other words, these variables work together in a sequential manner to mediate the impact of social media use on addiction. Additionally, the study indicates that social media use and social media addiction are fully mediated by both positive and negative affect, fear of missing out, and offline and online self-presentation.

**Discussion:**

Social media use can evoke both positive and negative affect. The longer individuals are immersed in social media, the more their positive affect intensifies, exacerbating FoMO and fostering inconsistent offline and online self-presentation. Conversely, extended social media engagement can heighten negative affect, leading to anxiety about others having more rewarding experiences and concurrently inducing apprehension characterized by FoMO. To prevent addiction, the development of educational tools such as SimCity video games, scenario-based learning activities, and virtual reality experiences focused on social media use and social media addiction can offer a valuable opportunity for pre-exposure to the related risks and challenges.

## 1 Introduction

Social media provides users with opportunities to exchange information and opinions, explore interests, communicate with friends, and grow one’s social network by connecting with a diverse group of people (Withington and Punch, 2019). Individuals use social media platforms, including collaborative projects, blogs, online communities for sharing content, social networking websites, virtual gaming worlds, and virtual social worlds (Kaplan and Haenlein, 2010), for diverse aims.

Social media use (SMU) has reached a staggering milestone, with approximately 5.17 billion individuals, almost 64% of the global population, actively engaging with these various platforms worldwide as of March 2024 (Shewale, 2024). This overview of the situation highlights the widespread adoption and pervasive influence of social media in today’s interconnected society.

Over recent decades, social media has become an integral part of people’s lives, particularly when they rely on technology to connect with others worldwide and access information at home and elsewhere, and the majority of research has focused on the negative factors of social media (e.g., Choi and Noh, 2019; El Abiddine et al., 2022). For example, studies have shown that more time spent using social media increases negative emotions (e.g., addiction, depression, loneliness, and anxiety) and fear of missing out (FoMO) (Houghton et al., 2018; Hunt et al., 2018; Shabahang et al., 2023), while increasing SMU has shown detrimental effects on the mental and physical well-being of both children and adults (Brown, 2021; Marengo et al., 2022).

By contrast, comparatively positive factors, especially SMU’s generation of positive emotions among users, including satisfaction, pride, and a sense of belonging (McKinsey Health Institute, 2023; Ng and Indran, 2023; Ostic et al., 2021), have been underexamined. Such positive emotions can enhance users’ self-disclosure as well as foster connection and enhance communication (Kim et al., 2014; Winstone et al., 2021). Notwithstanding these scarce findings, the potential beneficial impacts of SMU demand further examination.

Another important concept in this discussion is social media addiction (SMA), which is employed in this study to elucidate and characterize problematic SMU patterns across social networking sites (Andreassen, 2015; Andreassen et al., 2016). SMA, characterized by the compulsive and excessive use of social media platforms, is often considered a psychological disorder. Research has consistently shown that the symptoms of SMA share similarities with substance abuse disorders, including social anxiety, depression, and withdrawal symptoms (Bányai et al., 2017).

Drawing from the diagnostic criteria for pathological gambling outlined in the DSM-IV-TR, Young (1998) was the first to introduce the concept of Internet Addiction Disorder (IAD), which includes such characteristics as tolerance, preoccupation, and difficulty in reducing usage. IAD is known as problematic or pathological Internet use, which is only considered for the DSM-5-TR. However, the DSM-5-TR and International Classification of Diseases (ICD-11) have no longer classified excessive Internet use as a recognized disorder (American Psychiatric Association, 2022; World Health Organization, 2022). Instead of being considered a formal disorder, it is included only in the appendix of DSM-5-TR given the insufficient empirical evidence supporting Internet addiction (American Psychiatric Association, 2022: Murali and George, 2006).

SMA, known as problematic SMU (D’Arienzo et al., 2019; Lin et al., 2024), is characterized as a behavioral addiction marked by excessive concerns about social media, driven by an uncontrollable urge to engage with or access social platforms, and spending too much time and effort that it interferes with other important aspects of one’s life. However, it is controversial to use the term “addiction” in relation to social media and its potential for addiction (Domoff et al., 2022; Lin et al., 2024). Building upon this claim, it is necessary to expand the body of knowledge on SMA.

Individuals who use social media for more than two hours per day are twice as likely to suffer social isolation as those with an SMU of less than half an hour per day (Primack et al., 2017). Many individuals’ uncontrollable desire to browse social media pages results in high engagement in online activities, which can be linked to negative consequences in the personal, professional/academic, and social domains, including addiction, loneliness, depression, work impairment, low self-esteem, a diminished capacity to form meaningful relationships, poor sleep quality, poor well-being, and underperformance at work (Arrivillaga et al., 2022; Bányai et al., 2017; Lin et al., 2016, 2024; Zarate et al., 2023). Consequently, excessive engagement with social media can contribute to the development of SMA, which significantly impacts psychological well-being (Sun and Zhang, 2021).

### 1.1 Social media use and social media addiction

In this digital age, individuals tend to concentrate their attention on social media platforms, actively engaging with the diverse content and interactions offered. This sustained focus involves the allocation of cognitive resources and time in browsing, posting, liking, and responding through an ever-expanding stream (e.g., posts, photos, videos, and stories). A virtual environment offers the ability to remain anonymous (or at least non-identifiable) to dyadic or group interaction partners as well as find interaction partners who share aspects of one’s online self (Marriott and Buchanan, 2014). Social media platforms allow individuals to update, share, and publish content in various ways, enabling them to construct ideal and diverse self-presentations (Nadkarni and Hofmann, 2012). The captivating allure of visually appealing informative and interactive elements integrated within social media platforms contribute to individuals’ enjoyment and satisfaction, fulfilling their psychological needs for self-expression, social connection, and content discovery. Nevertheless, this flow experience can also be characterized by intensified concentration, a gradual loss of self-awareness, and a merging of action and consciousness. It is also accompanied by a strong sense of control, a distorted perception of time, a feeling of relief, and a high level of intrinsic reward (Csikszentmihalyi, 1990). Such excessive concentration and pleasure may lead to SMA.

*Hypothesis 1*: SMU positively predicts SMA (H1).

### 1.2 Positive and negative affect

Positive affect refers to the inclination to encounter positive emotions and engage in positive interactions with others despite life’s difficulties, indicating the level of enthusiasm, activity, and alertness. High positive affect signifies a state of heightened energy, complete focus, and enjoyable involvement (Li et al., 2020; Watson et al., 1988). By contrast, negative affect refers to perceiving the world in a negative manner and encompasses various aversive emotions such as anger, contempt, disgust, guilt, fear, and nervousness (Malodia et al., 2024). However, when expressed in the wrong situations, emotions tend to be harmful.

Flow experience can occur in both positive and negative emotional states, encompassing a wide range of emotional arousal (Brock, 2017). It is more likely to manifest when individuals are experiencing positive emotions (Csikszentmihalyi, 2014). Individuals engaged and immersed in activities tend to exhibit persistent behavioral involvement when accompanied by positive emotions (Skinner and Belmont, 1993). When individuals experience flow, they often describe being “in the zone” or feeling completely absorbed in the present moment. Individuals experience positive affect when engaging in pleasurable and exciting SMU, especially during the early stages of use, as they communicate with others and participate in various activities.

Nevertheless, individuals may experience negative emotions, including irritability, anxiety, and depression, as a result of excessive SMU, leading to the development of SMA. For instance, peer exclusion and rejection on social media can be distressing for adolescents, compromising feelings of belonging, competency, and autonomy, which can threaten their achievement of developmental tasks (Marengo et al., 2021; Zimmer-Gembeck, 2016). However, the impact of negative affect on flow experience merits further scholarly investigation.

*Hypothesis 2*: Positive affect (H_2a_) and negative affect (H_2b_) act as mediators between SMU and SMA.

### 1.3 FoMO

FoMO is the pervasive fear that others may be enjoying rewarding experiences while one is missing out (Przybylski et al., 2013a), leading individuals to have a strong desire to stay updated on the statuses of others, even when they believe that others are experiencing satisfaction in their absence (Błachnio and Przepiórka, 2018). FoMO, which is strongly associated with high SMU (Dinçer et al., 2022), leads to the performance of continuous online activities (Cao et al., 2018; Rosen et al., 2018) and other adverse behaviors (Stead and Bibby, 2017). Studies have also found that SMU affects FoMO and has long-lasting consequences on individuals’ psychological well-being (Baker et al., 2016; Marengo et al., 2021).

The abundance of hedonic gratifications provided by social media platforms has led to protracted engagement among users. The availability of diverse forms of social interaction and entertainment contributes to the allure of these platforms. Moreover, the reinforcement loop created by repeated exposure to gratifications fosters profound conditional behavior. Excessive SMU engenders compulsive checking behaviors, wherein individuals are predisposed to habitually inspecting their social media accounts in a state of unawareness. These compulsive disorders have been termed “disconnection syndrome” and “ring or phantom vibration syndrome” (Drouin et al., 2012).

The negative emotions arising from being exposed to unfavorable content and information on social media can intensify FoMO and lead to frequent engagement to avoid missing others’ updates as well as concern about being overlooked. Individuals may experience agitation, anxiety, and unease when perceiving themselves as excluded by their peers. FoMO is often considered an antecedent of negative behaviors such as SMA and problematic Internet use (Fang et al., 2020; Gori et al., 2023; Servidio et al., 2024) as well as inauthentic self-presentation on social networking sites (Wang et al., 2018). Feelings of anxiety or alienation increase SMU as a means to alleviate the fear of isolation and to seek relief. Driven by these compensatory mechanisms, individuals are negatively reinforced and potentially become addicted to social media (Monacis et al., 2021).

*Hypothesis 3*: FoMO acts as a mediator between SMU and SMA (H_3_).

### 1.4 Offline and online self-presentation

Self-presentation is a behavioral trait where individuals intentionally build their image to create a desired impression on others (Fox and Vendemia, 2016). Individuals can be deliberate about how they present themselves online; for example, they can intentionally post specific content, edit existing information, and delete content that shows them in a less favorable way (Fullwood et al., 2020). In particular, social media allows people to experiment with various self-presentations and see how others react. By receiving approval (e.g., likes and comments), a particular self-presentation can be authenticated, which may then be incorporated into one’s offline identity (Manago et al., 2008).

Zhao et al. (2008) found that emerging adults use specific strategies to present their online self on Facebook. However, other studies show consistency between offline and online self-presentation despite omitting or exaggerating certain aspects of themselves (Fullwood et al., 2016; Marriott and Buchanan, 2014; Strimbu and O’Connell, 2019). Individuals who present a false ideal self are less affected psychologically by the reality of the situation and disappointment than those who think they are presenting their real selves to others online (Attrill-Smith, 2019). Consequently, this behavior can increase the risk of SMA among certain individuals.

*Hypothesis 4*: Offline and online self-presentation acts as a mediator between SMU and SMA (H_4_).

Human psychological development and emotional suffering are adversely affected by negative experiences (e.g., FoMO) in SMU, particularly in the early stages of adolescence (Marengo et al., 2021). Studies have found that individuals experiencing depression exhibit specific patterns of SMU, including perceiving themselves in an idealistic manner compared with others, sharing risky behaviors, posting negative or harmful content, and engaging in negative verbal interactions with others (Radovic et al., 2017). Additionally, excessive SMU is associated with negative impacts such as FoMO, multiple online self-presentation, and addiction (Al-Busaidi et al., 2023; Fullwood et al., 2016; Talan et al., 2024; Wang, 2021; Wolniewicz et al., 2018). However, the impact of excessive SMU on individuals has not been fully explored, particularly its interrelations with positive and negative affect, FoMO, online self-presentation, and SMA. Hence, the interconnectedness of these factors and their role as serial multiple mediators in the relationship between SMU and SMA need to be investigated.

*Hypothesis 5*: Positive affect and FoMO (H_5a_) as well as negative affect and FoMO (H_5b_) act as serial mediators between SMU and SMA.

*Hypothesis 6*: Positive affect and offline and online self-presentation (H_6a_) as well as negative affect and offline and online self-presentation (H_6b_) act as serial mediators between SMU and SMA.

*Hypothesis 7*: FoMO and offline and online self-presentation act as serial mediators between SMU and SMA (H_7_).

*Hypothesis 8*: Positive affect, FoMO, and offline and online self-presentation (H_8a_) as well as negative affect, FoMO, and offline and online self-presentation (H_8b_) act as serial mediators between SMU and SMA.

**Figure 1:**
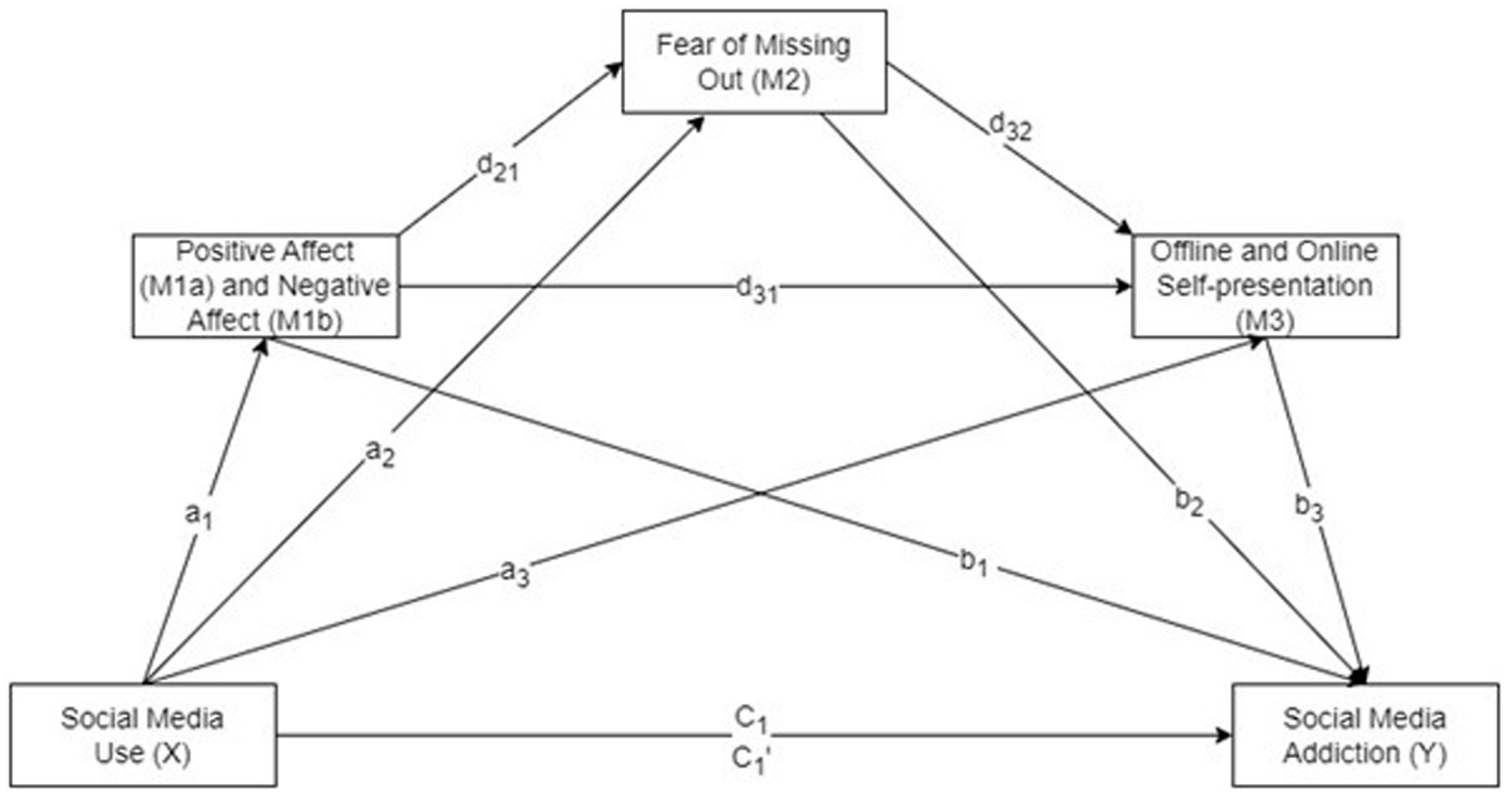
The hypothesized serial multiple mediation model with three mediators (M_1a_ = positive affect, M_1b_ = negative affect, M_2_ = fear of missing out, M_3_ = offline and online self-presentation). Positive affect and negative affect were tested separately.

Based on the foregoing, this cross-sectional study investigates the serial mediating effects of positive and negative affect, FoMO, and offline and online self-presentation on the relationship between SMU and SMA, providing insights into the psychological impacts of SMU. Figure 1 illustrates the hypothesized serial multiple mediation model.

## 2 Materials and methods

### 2.1 Participants

#### 2.1.1 Recruitment feasibility

Power analysis was performed using the G*Power 3.0.10 program to determine the required sample size for the study. Accordingly, with a power level of 0.95 and a significance level of 0.05, a small effect size of *r* = 0.20 (Cohen, 1988) was determined. Based on the analysis, the required sample size was 385.

To be eligible for inclusion, participants (*N =* 385, age range = 18–60; *M* = 25.37 years; *SD* = 8.61) had to report using social media. Women were overrepresented (*n* = 324, 84.2%) compared with men (*n* = 61, 15.8%). Most belonged to Gen Z (*n* = 306, 79.5%). The majority had completed a bachelor’s degree (*n* = 151, 39.2%) and sub-degree programs (*n* = 130, 33.8%). More than half of the participants were working in a part-time job (*n* = 208, 54%). Table 1 summarizes the participants’ characteristics.

**Table 1:** Participants’ characteristics (*N* = 385).

		*N*	%
Gender	Male	61	15.8
	Female	324	84.2
Age (years)	12–27 (Generation Z)	306	79.5
	28–43 (Millennials)	54	14.0
	44–59 (Generation X)	24	6.2
	60–69 (Boomers II)	1	0.3
Educational attainment	Secondary school	2	0.5
	High school/university-preparatory school	82	21.3
	Sub-degree (e.g., certificate, higher diplomas, diplomas)	130	33.8
	Bachelor’s degree	151	39.2
	Master’s, postgraduate diploma, postgraduate certificate, postgraduate diploma in education	18	4.7
	PhD	2	0.5
Employment status	Working full-time	75	19.5
	Working part-time	208	54.0
	Retired	1	0.3
	Unemployed	3	0.8
	Not employed	77	20.0
	Student	21	5.4

### 2.2 Measures

#### 2.2.1 SMU

The following 10 items captured SMU: (1) time spent on social media daily; (2) types of social media platforms used (e.g., Facebook, Instagram); (3) types of social media platforms most often used; (4) social media functions most often used (e.g., instant messaging, social networking); (5) functions of AI-generated content (e.g., AI-generated content/writing, image creation with AI/augmented reality filter); (6) social media content most often browsed (e.g., pictures, text, stories); (7) the frequency of updating your status/sharing or publishing posts, photos, videos, or text on social media; (8–10) and the frequencies of browsing, liking, and responding to others’ status updates, posts, photos, videos, and text on social media. Items (7–10) were answered on a six-point Likert scale (1 = *very rarely/never*, 6 = *several times a day*), with higher scores indicating a higher frequency of SMU. The SMU scale had a Cronbach’s alpha coefficient of 0.75 in this study.

#### 2.2.2 Positive and Negative Affect Schedule

The Positive and Negative Affect Schedule (Watson et al., 1988) measures positive and negative affect over the past week. It consists of 20 items rated on a five-point Likert scale (1 = *very slightly or not at all*, 5 = *extremely*), divided into two 10-item subscales related to positive affect (e.g., “interested”) and negative affect (e.g., “distressed”). Higher scores represent higher levels of positive affect, while lower scores represent lower levels of negative affect. The Cronbach’s alphas for the two subscales ranged from 0.87 to 0.95, with the overall scale having a Cronbach’s alpha of 0.93 in this study.

#### 2.2.3 Fear of Missing Out Scale

The Fear of Missing Out Scale (Przybylski et al., 2013a, 2013b) assesses participants’ FoMO via their everyday experiences. The scale has 10 items (e.g., “I get anxious when I do not know what my friends are up to”) rated on a five-point Likert scale (1 = *not at all true of me*, 5 = *extremely true of me*). Higher scores indicate a higher level of FoMO. The total scale had a Cronbach’s alpha of 0.89 in this study.

#### 2.2.4 Presentation of Online Self Scale

The Presentation of Online Self Scale (Fullwood et al., 2016) assesses participants’ experiences of offline and online self-presentation. It consists of 21 items rated on a five-point Likert scale (1 = *strongly disagree*, 5 = *strongly agree*). The scale comprises four subscales: ideal self (e.g., “I can show my best qualities online”), multiple selves (e.g., “I enjoy acting out different identities online”), consistent self (e.g., “I feel I am the same person in the cyber world that I am in the real world”), and online presentation preference (e.g., “I find it difficult to be myself in the real world”); these subscales have nine, five, four, and three items, respectively. Higher scores indicate higher presentation. Given the risk of the discrepancy between one’s ideal self and true self, this study investigated the ideal self, multiple selves, and online presentation preference subscales. The Cronbach’s alphas for these subscales ranged from 0.64 to 0.92 and the overall scale had a Cronbach’s alpha of 0.86 in this study.

#### 2.2.5 Bergen Social Media Addiction Scale

The Bergen Social Media Addiction Scale (Andreassen et al., 2016) assesses experiences with addictive behaviors in the previous 12 months. It consists of six items (e.g., “You feel an urge to use social media more and more”) rated on a five-point Likert scale (1 = *very rarely*, 5 = *very often*). Higher scores indicate a greater risk of problematic SMU. The scale had a Cronbach’s alpha of 0.82 in this study.

### 2.3 Data collection and analysis

The participants were recruited via three of the most popular social media platforms (i.e., Facebook, Instagram, and WhatsApp) by convenience sampling. A link or QR code with an overview of the study was provided via a self-administered online survey, which took approximately 20 min. The online survey was divided into five parts: an introductory page, an informed consent statement, SMU habits, questionnaires of the four scales, and demographic questions. The online survey offered an acceptable degree of privacy and anonymity. IBM SPSS 29 was used for quantitative analyses. There were no missing data. To examine the relationships between the variables in the serial multiple mediation analyses, bivariate correlations were computed. Harman’s single-factor test was used to check that common method bias was not present (Podsakoff et al., 2003). All the variables were included in an exploratory factor analysis and the unrotated factor solution was examined (McFarlin and Sweeney, 1992).

Sequential mediation models were analyzed to investigate the hypotheses. To ascertain the indirect effects, 10,000 bootstrap samples were employed in conjunction with Model 6 of the PROCESS macro in SPSS (version 4.3.1; Hayes, 2022). Using the bootstrapping technique of Bollen and Stine (1990) yielded 95% bias-corrected bootstrap confidence intervals. Statistical significance was set at 5% (Cohen et al., 2016).

### 2.4 Ethical considerations

This study followed the ethical guidelines and standards set forth by the American Psychological Association and received approval from the university’s Research Ethics Committee after a thorough review. The primary data remained confidential and the participants’ identities were protected, as only aggregated findings were presented, without disclosing individual details in the analysis. All the participants were fully informed about the aims of the study and informed consent was obtained. There was a clear statement that their involvement would not cause them any harm, either physically or psychologically. Further, the participants were informed that they could withdraw from the study at any time without incurring any consequences.

## 3 Results

### 3.1 Descriptive statistics

As presented in Table 2, all the variables were significantly correlated (Cohen, 1988). SMU had a weak, positive, and statistically significant correlation with positive affect (*r* = 0.11, *p* < 0.05), negative affect (*r* = 0.18, *p* < 0.001), FoMO (*r* = 0.13, *p* < 0.05), offline and online self-presentation (*r* = 0.17, *p* < 0.01), and SMA (*r* = 0.14, *p* < 0.01). Positive affect had a moderate, positive, statistically significant correlation with negative affect (*r* = 0.58, *p* < 0.001), FoMO (*r* = 0.39, *p* < 0.001), offline and online self-presentation (*r* = 0.51, *p* < 0.001), and SMA (*r* = 0.49, *p* < 0.001). Negative affect had a moderate, positive, statistically significant correlation with FoMO (*r* = 0.42, *p* < 0.001), offline and online self-presentation (*r* = 0.47, *p* < 0.001), and SMA (*r* = 0.47, *p* < 0.001). FoMO had a moderate, positive, statistically significant correlation with offline and online self-presentation (*r* = 0.51, *p* < 0.001) and a strong, positive, and statistically significant correlation with SMA (*r* = 0.62, *p* < 0.001). Offline and online self-presentation had a moderate, positive, and statistically significant correlation with SMA (*r* = 0.59, *p* < 0.001).

**Table 2:** Means, standard deviations, and correlations among the variables (*N* = 385).

Variable	*M*	*SD*	1	2	3	4	5
1. SMU	5.49	3.72					
2. Positive affect^@^	25.70	6.89	0.11*				
3. Negative affect^@^	19.36	7.71	0.18***	0.58***			
4. FoMO^^^	23.67	7.83	0.13*	0.39***	0.42***		
5. Offline and online self-presentation^#^	42.46	11.57	0.17**	0.51***	0.47***	0.51***	
6. SMA©	14.42	4.70	0.14**	0.49***	0.47***	0.62***	0.59***

^@^Positive and Negative Affect Schedule; ^^^Fear of Missing Out Scale; ^#^Presentation of Online Self Scale without consistent self; ©Bergen Social Media Addiction Scale. Values on the diagonal are the Cronbach’s α coefficients. *p < 0.05, **p < 0.01, ***p < 0.001.

### 3.2 Serial multiple mediation analyses

**Figure 2:**
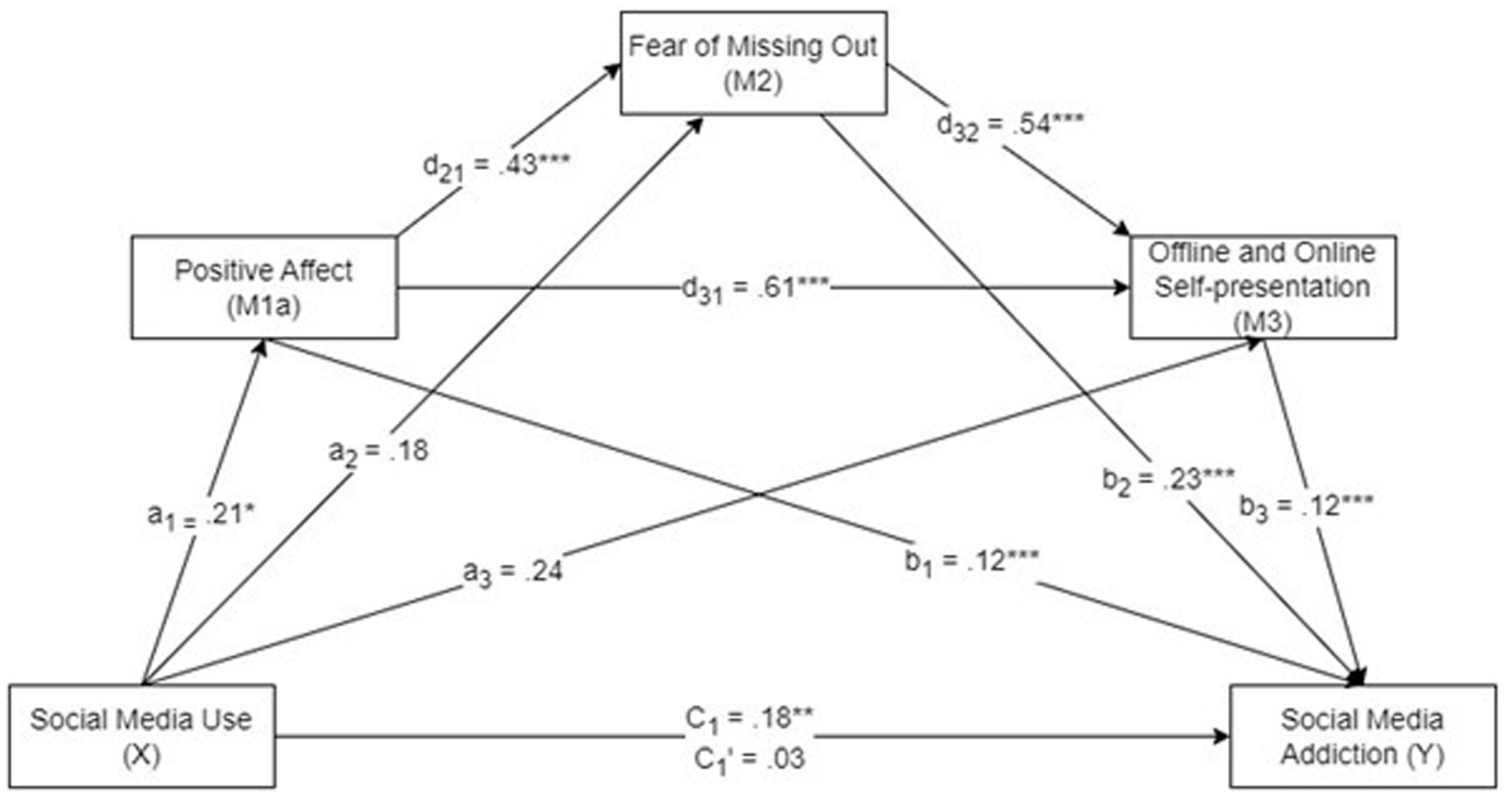
The serial multiple mediation analysis of the relationship between social media use and social media addiction mediated via positive affect, fear of missing out, and offline and online self-presentation. c: total effect of SMU; c’: direct effect of social media use with the mediators controlled for. Unstandardized regression coefficients are reported. *p < 0.05, **p < 0.01. ***p < 0.001.

**Figure 3:**
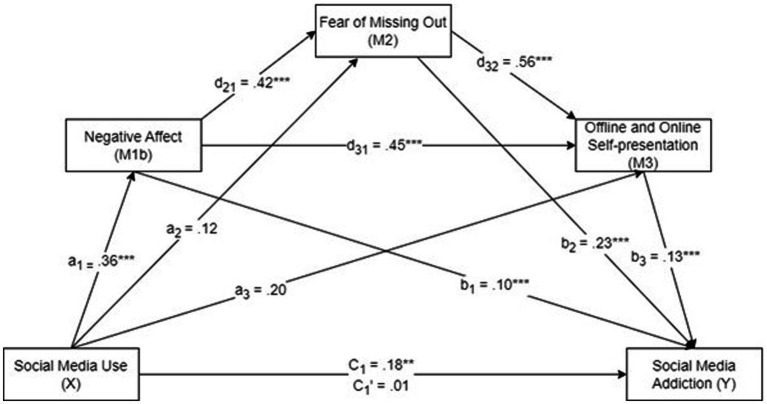
The serial multiple mediation analysis of the relationship between social media use and social media addiction mediated via negative affect, fear of missing out, and offline and online self-presentation. c: total effect of SMU; c’: direct effect of SMU with the mediators controlled for. Unstandardized regression coefficients are reported. **p < 0.01, ***p < 0.001.

Table 3 summarizes the results of the first PROCESS analysis. Figures 2, 3 show the results of the serial multiple mediation analyses.

**Table 3:** Summary of the multiple regression analyses for the serial multiple mediation model.

Variable	Model 1					Model 2					Model 3					Model 4				
	*B*	95% CI	*SE B*	*β*	*t*	*B*	95% CI	*SE B*	*β*	*t*	*B*	95% CI	*SE B*	*β*	*t*	*B*	95% CI	*SE B*	*β*	*t*
Constant	24.56	[23.34, 25.79]	0.62		39.42***	11.55	[8.66, 14.44]	1.47		7.85***	12.87	[8.92, 16.82]	2.01		6.40***	0.39	[−1.12, 1.90]	0.77		0.51
SMU	0.21	[0.02, 0.39]	0.09	0.11	2.22*	0.18	[−0.01, 0.38]	0.10	0.09	1.85	0.24	[−0.01, 0.49]	0.13	0.07	1.91	0.03	[−0.06, 0.12]	0.05	0.02	0.57
Positive affect						0.43	[0.33, 0.54]	0.05	0.38	8.06***	0.61	[0.46, 0.75]	0.07	0.36	8.26***	0.12	[0.06, 0.18]	0.03	0.18	4.12***
FoMO											0.53	[0.41, 0.66]	0.06	0.36	8.22***	0.23	[0.18, 0.89]	0.03	0.39	9.17***
Offline and online self-presentation																0.12	[0.09, 0.16]	0.02	0.31	6.73***
	*R*^2^ = 0.01, *F*_(1, 383)_ = 4.91, *p* < 0.05					*R*^2^ = 0.16, *F*_(2, 382)_ = 36.29, *p* < 0.001					*R*^2^ = 0.38, *F*_(3, 381)_ = 78.83, *p* < 0.001					*R*^2^ = 0.51, *F*_(4, 380)_ = 99.55, *p* < 0.001				

*N* = 385. Model 1, predictor variable: SMU; outcome variable: positive affect. Model 2, predictor variables: SMU and positive affect; outcome variable: FoMO. Model 3, predictor variables: SMU, positive affect, and FoMO; outcome variable: offline and online self-presentation. Model 4, predictor variables: SMU, positive affect, FoMO, and offline and online self-presentation; outcome variable: SMA. SE, standard error. *p < 0.05, ***p < 0.001.

### 3.3 Mediating roles of positive affect, FoMO, and offline and online self-presentation

The results showed a total effect (c) of SMU on SMA through the mediators, *B* = 0.18, *p* < 0.01. This finding indicated that increased SMU generated a greater degree of SMA with the mediators. However, when the mediators were removed from the analysis, this coefficient was not statistically significant (direct effect, c’), *B* = 0.03, *p* = 0.57, rejecting H_1_. SMU was also found to be a positive predictor of positive affect (*B* = 0.21, *p* < 0.05) (a_1_), but not a predictor of FoMO (*B* = 0.18, *p* = 0.66) (a_2_) or offline and online self-presentation (*B* = 0.24 *p* = 0.07) (a_3_) (Figure 2).

As shown in Table 4, the analysis of the indirect mediating effects by bootstrapping found that the resulting data supported the significance of Path 1 (SMU ➔ Positive affect ➔ SMA; *B* = 0.0248, *SE* = 0.0159, 95% CI [0.0030, 0.0641]), confirming H_2a_; Path 3 (SMU ➔ FoMO ➔ SMA; *B* = 0.0303, *SE* = 0.0202, 95% CI [0.0009, 0.0797]), confirming H_4_; Path 4 (SMU ➔ Positive affect ➔ FoMO ➔ SMA; *B* = 0.0210, *SE* = 0.0134, 95% CI [0.0026, 0.0555]), confirming H_5a_; Path 5 (SMU ➔ Positive affect ➔ Offline and online self-presentation ➔ SMA; *B* = 0.0158, *SE* = 0.0099, 95% CI [0.0020, 0.0405]), confirming H_6a_; and Path 7 (SMU ➔ Positive affect ➔ FoMO ➔ Offline and online self-presentation ➔ SMA; *B* = 0.0060, *SE* = 0.0038, 95% CI [0.0008, 0.0155]), confirming H_8a_. However, Paths 2 and 6 were not significant, rejecting H_3_ and H_7_, respectively.

**Table 4:** Bootstrapping indirect effects and 95% CI for sequential mediation model 1.

Model pathway	Effect	Boot SE	95% CI	
			Lower	Upper
Path 1 SMU ➔ Positive affect ➔ SMA	0.0248	0.0159	0.0030	0.0641
Path 2 SMU ➔ FoMO ➔ SMA	0.0429	0.0242	−0.0055	0.0920
Path 3 SMU ➔ Offline and online self-presentation ➔ SMA	0.0303	0.0202	0.0009	0.0797
Path 4 SMU ➔Positive affect ➔ FoMO ➔ SMA	0.0210	0.0134	0.0026	0.0555
Path 5 SMU ➔Positive affect ➔ Offline and online self-presentation ➔ SMA	0.0158	0.0099	0.0020	0.0405
Path 6 SMU ➔ FoMO ➔ Offline and online self-presentation ➔ SMA	0.0122	0.0074	−0.0014	0.0281
Path 7 SMU ➔Positive affect ➔ FoMO ➔ Offline and online self-presentation ➔ SMA	0.0060	0.0038	0.0008	0.0155
Total	0.1530	0.0557	0.0681	0.2823

CI, Confidence interval; 10,000 bootstrap samples with 95% CI.

The results revealed a significant indirect effect of SMU on SMA through positive affect, FoMO, and offline and online self-presentation (*b* = 0.0060, *t* = 1.5789), supporting H_8a_. Furthermore, the total effect of SMU on SMA in the presence of the mediators was significant (*b* = 0.1792, *p* < 0.01). Hence, positive affect, FoMO, and offline and online self-presentation fully mediated the relationship between SMU and SMA. Table 4 summarizes the mediation model results.

### 3.4 Mediating roles of negative affect, FoMO, and offline and online self-presentation

Table 5 summarizes the results of the second PROCESS analysis. The results showed a total effect (c) of SMU on SMA through the mediators, *B* = 0.18, *p* < 0.01. This finding indicated that increased SMU generated a greater degree of SMA with the mediators. However, when the mediators were removed from the analysis, this coefficient was not statistically significant (direct effect, c’), *B* = 0.01, *p* = 0.77, rejecting H_1_. SMU was also found to be a positive predictor of negative affect (*B* = 0.36, *p* < 0.001) (a_1_), but not a predictor of FoMO (*B* = 0.12, *p* = 0.22) (a_2_) or offline and online self-presentation (*B* = 0.20, *p* = 0.13) (a_3_) (Figure 3).

**Table 5:** Summary of the multiple regression analyses for the serial multiple mediation model.

Variable	Model 1					Model 2					Model 3					Model 4				
	*B*	95% CI	*SE B*	*β*	*t*	*B*	95% CI	*SE B*	*β*	*t*	*B*	95% CI	*SE B*	*β*	*t*	*B*	95% CI	*SE B*	*β*	*t*
Constant	17.36	[16.01, 18.72]	0.69		25.15***	14.86	[12.80, 16.93]	1.05		14.15***	19.47	[16.09, 22.85]	1.72		11.33***	1.43	[0.51, 2.81]	0.70		2.04*
SMU	0.36	[0.16, 0.57]	0.10	0.18	3.49***	0.12	[−0.07, 0.31]	0.10	0.06	1.22	0.20	[−0.06, 0.46]	0.13	0.06	1.52	0.01	[−0.08, 0.11]	0.05	0.01	0.30
Negative affect						0.42	[0.33, 0.51]	0.05	0.41	8.81***	0.45	[0.31, 0.59]	0.07	0.30	6.47***	0.09	[0.04, 0.14]	0.03	0.15	3.55***
FoMO											0.56	[0.42, 0.69]	0.07	0.38	8.22***	0.23	[0.18, 0.28]	0.03	0.38	8.84***
Offline and online self-presentation																0.13	[0.10, 0.17]	0.02	0.33	7.43***
	*R*^2^ = 0.03, *F*_(1, 383)_ = 12.20, *p* < 0.001					*R*^2^ = 0.18, *F*_(2, 382)_ = 42.79, *p* < 0.001					*R*^2^ = 0.34, *F*_(3, 381)_ = 66.74, *p* < 0.001					*R*^2^ = 0.51, *F*_(4, 380)_ = 97.42, *p* < 0.001				

*N* = 385. Model 1, predictor variable: SMU; outcome variable: negative affect. Model 2, predictor variables: SMU and negative affect; outcome variable: FoMO. Model 3, predictor variables: SMU, negative affect, and FoMO; outcome variable: offline and online self-presentation. Model 4, predictor variables: SMU, negative affect, FoMO, and offline and online self-presentation; outcome variable: SMA. SE, standard error. *p < 0.05. ***p < 0.001.

As presented in Table 6, the analysis of the indirect mediating effects by bootstrapping found that the resulting data supported the significance of Path 1 (SMU ➔ Negative affect ➔ SMA; *B* = 0.0349, *SE* = 0.0158, 95% CI [0.0110, 0.0729]), confirming H_2b_; Path 4 (SMU ➔ Negative affect ➔ FoMO ➔ SMA; *B* = 0.0355, *SE* = 0.0135, 95% CI [0.0163, 0.0685]), confirming H_5b_; Path 5 (SMU ➔ Negative affect➔ Offline and online self-presentation ➔ SMA; *B* = 0.0204, *SE* = 0.0088, 95% CI [0.0078, 0.0421]), confirming H_6b_; and Path 7 (SMU ➔ Negative affect ➔ FoMO ➔ Offline and online self-presentation ➔ SMA; *B* = 0.0112, *SE* = 0.0047, 95% CI [0.0046, 0.0229]), confirming H_8b_. However, Paths 2, 3, and 6 were not significant, rejecting H_3_, H_4_, and H_7_, respectively.

**Table 6:** Bootstrapping indirect effects and 95% CI for sequential mediation model 2.

Model pathway	Effect	Boot SE	95% CI	
			Lower	Upper
Path 1 SMU ➔ Negative affect ➔ SMA	0.0349	0.0158	0.0110	0.0729
Path 2 SMU ➔ FoMO ➔ SMA	0.0279	0.0236	−0.0187	0.0762
Path 3 SMU ➔ Offline and online self-presentation ➔ SMA	0.0241	0.0211	−0.0076	0.0785
Path 4 SMU ➔ Negative affect ➔ FoMO ➔ SMA	0.0355	0.0135	0.0163	0.0685
Path 5 SMU ➔ Negative affect ➔ Offline and online self-presentation ➔ SMA	0.0204	0.0088	0.0078	0.0421
Path 6 SMU ➔ FoMO ➔ Offline and online self-presentation ➔ SMA	0.0088	0.0074	−0.0057	0.0243
Path 7 SMU ➔ Negative affect ➔ FoMO ➔ Offline and online self-presentation ➔ SMA	0.0112	0.0047	0.0046	0.0229
Total	0.1628	0.0541	0.0790	0.2899

10,000 bootstrap samples with 95% CI.

The results revealed a significant indirect effect of SMU on SMA through negative affect, FoMO, and offline and online self-presentation (*b* = 0.0115, *t* = 2.3958), supporting H_8b_. Furthermore, the total effect of SMU on SMA in the presence of the mediators was significant (*b* = 0.1792, *p* < 0.01). Hence, negative affect, FoMO, and offline and online self-presentation fully mediated the relationship between SMU and SMA. Table 6 summarizes the mediation model results. This relationship was mediated only by negative affect, but not FoMO or offline and online self-presentation. These findings reconfirmed those of previous studies that the duration of SMU is positively related to SMA by enhancing negative affect, FoMO, and offline and online self-presentation (Radovic et al., 2017; Wolniewicz et al., 2018).

## 4 Discussion

A serial multiple mediation model was used to examine how positive and negative affect, FoMO, and offline and online self-presentation mediated the relationship between SMU and SMA. Significant correlations were found among all the variables. In the serial multiple mediation analyses, SMU had no direct effect on SMA, inconsistent with the findings of prior studies (e.g., Sun and Zhang, 2021; Talan et al., 2024) that have shown that SMU is a significant predictor of SMA.

These findings raise concerns about the exclusion of IAD from the DSM-5-TR; as noted above, owing to insufficient empirical evidence, it was included only in the appendix of DSM-5-TR instead of being considered a formal disorder (American Psychiatric Association, 2022; World Health Organization, 2022). Hence, excessive SMU, though considered unhealthy, is no longer formally categorized as an addiction or disorder, leading to a lack of consensus on this behavior (Wegmann et al., 2020). Specifically, SMU can promote feelings of happiness among users (Pittman, 2018). However, individuals addicted to the virtual world have trouble communicating face-to-face; spend less time with their family, friends, acquaintances, and immediate surroundings; and can suffer mood disorders (e.g., dissatisfaction and stress) (Geçikli, 2020; Hussain and Griffiths, 2021). The total effect of SMU on SMA was significant and their relationship was mediated by positive affect, but not by FoMO or offline and online self-presentation. These findings reaffirm and broaden research on the relationship between positive affect and SMA (Wang, 2021). Specifically, the longer users engage with social media, the higher positive affect, which exacerbates FoMO and promotes inconsistent offline and online self-presentation. These factors collectively contribute to the manifestation and progression of SMA (American Psychiatric Association, 2022; World Health Organization, 2022).

Similarly, SMU and SMA were mediated by negative affect, but not by FoMO or offline and online self-presentation, consistent with a previous finding that individuals exhibit elevated negative affect when engaging with social media (Hunt et al., 2018). The longer individuals engage with social media, the higher negative affect, resulting in anxiety that others are having more rewarding experiences, and concurrently experiencing apprehension characterized by FoMO. Previous studies have shown that social media users have varying motives and expectations from social media, including its ability to regulate negative emotions, fulfill social needs, and provide positive feedback from others (Krämer and Winter, 2008; Neubaum and Krämer, 2015). This sense of comparative evaluation increases the tendency to present an inconsistent self-presentation aligned with users’ desired self-image, raising the inclination to use social media excessively. Consequently, this excessive usage can lead to the development of SMA.

As hypothesized, the results showed that the relationship between SMU and SMA was mediated by positive and negative affect as well as FoMO and offline and online self-presentation. These results provide support for our hypothesis that SMU has an elevated impact on both positive and negative affect. Wang (2021) found that satisfaction, a sense of pride, and a sense of belonging are the most influential positive factors contributing to social media overuse. Hence, to address SMA, it is crucial to not only focus on negative emotions but also pay attention to fostering these specific positive emotions.

### 4.1 Limitation

These are three potential limitations of the study. First, the predominantly female sample (84.2%) in this study may limit the generalizability of the findings, as women are often more emotionally expressive than men (Ciarrochi et al., 2005). A more balanced gender representation in future research, including recruiting individuals from different generations and geographic locations, could thus enhance generalizability. The lack of geographic borders allows the dissemination of social media information across locations and time zones. Hence, exploring the impact of FoMO in different regions would be a worthwhile future research avenue. Findings derived by involving participants of various ages and genders could better reflect and apply to a more extensive population.

Second, the present study solely relied on quantitative data. To better understand the underlying factors shaping positive and negative affect in relation to SMU, future research could employ a mixed-methods design that combines surveys with interviews. By incorporating qualitative and quantitative data, researchers could gather rich in-depth insights and narratives from social media users to shed light on the intricate relationship between SMU and its impact on affect.

Third, this research failed to differentiate between personal, professional, and academic SMU. Therefore, further investigation is needed into the impact of SMU on psychological and physiological health considering the specific contexts in which it is used. It is imperative to avoid the over-pathologization of everyday behaviors, while concurrently refraining from trivializing conditions that hold clinical importance and warrant public health consideration. To develop professional standards, the implementation of empirical-based studies could guide both the research endeavors and the clinical practices of psychiatrists and psychologists (Brand et al., 2020).

### 4.2 Implication

Notwithstanding these limitations, this study’s findings have three important implications. First, this study demonstrates the significance of positive and negative emotions, emphasizing the importance of positive and negative affect in the context of SMU. Individuals are inclined to share misinformation when it aligns with their personal identity or social norms, when it is novel, and when it elicits strong emotions. Research indicates that detecting misinformation is challenging, as individuals tend to focus on understanding and deciding what to do with new information, rather than critically evaluating its accuracy (American Psychological Association, 2023). By recognizing the influence of these affective states, researchers could develop effective strategies and interventions to mitigate the risk of SMA. The integration of verification and fact-checking mechanisms into the educational system could help prevent, recognize, and avoid the spread of fake news and misinformation.

Second, these insights could also inform the design of preventive measures and interventions that consider the impact of positive and negative affect on individuals’ psychological well-being in the context of SMU, alongside their other needs (e.g., cognitive, affective, personal integrative, social integrative, and tension-free needs). The development of educational tools such as SimCity videogames, scenario-based learning activities, and virtual reality experiences centered on SMU and SMA could provide the public with valuable pre-exposure to the associated risks and challenges. Subsequently, post-experience debriefing sessions could foster group reflection, offer recommendations, and suggest effective approaches through collaborative discussions.

Third, this study provides insights that can encourage individuals to find a healthy equilibrium between their online and offline activities and prevent the development of SMA. While the widespread adoption of social media has diminished the importance of geographic proximity, concern about the escalation of SMU leading to potential issues such as SMA is growing. Social media users often underestimate the severity of SMA due to a lack of awareness of its critical nature (Sun and Zhang, 2021). Unlike drug addiction, where evident consequences such as weight loss, financial loss, and strained relationships bring the issue to the forefront, SMA may be more challenging to acknowledge or accept due to the absence of such overt signs (Giordano, 2022). Despite the exclusion of IAD from the DSM-5-TR, studies must explore this phenomenon and better understand its implications. Further research in this area could shed light on the conceptualization and classification of problematic Internet use within the broader spectrum of mental health disorders.

## Data Availability

The original contributions presented in the study are included in the article/supplementary material, further inquiries can be directed to the corresponding author.

## References

[ref1] Al-Busaidi A. S. Dauletova V. Al-Wahaibi I. (2023). The role of excessive social media content generation, attention seeking, and individual differences on the fear of missing out: a multiple mediation model. Behav. Inf. Technol. 42, 1389–1409. doi: 10.1080/0144929X.2022.2075791

[ref2] American Psychiatric Association (2022). Diagnostic and statistical manual of mental disorders (5th ed, text revision, DSM-5-TR™). Washington, DC: American Psychiatric Association.

[ref3] American Psychological Association. (2023). Using psychological science to understand and fight health misinformation: An APA consensus statement. Available online at: https://www.apa.org/pubs/reports/misinformation-recommendations.pdf (Accessed November 19, 2024)

[ref4] Andreassen C. S. (2015). Online social network site addiction: a comprehensive review. Curr. Addict. Rep. 2, 175–184. doi: 10.1007/s40429-015-0056-9

[ref5] Andreassen C. S. Billieux J. Griffiths M. D. Kuss D. J. Demetrovics Z. Mazzoni E.. (2016). The relationship between addictive use of social media and video games and symptoms of psychiatric disorders: a large-scale cross-sectional study. Psychol. Addict. Behav. 30, 252–262. doi: 10.1037/adb0000160, PMID: 26999354

[ref6] Arrivillaga C. Rey L. Extremera N. (2022). A mediated path from emotional intelligence to problematic social media use in adolescents: the serial mediation of perceived stress and depressive symptoms. Addict. Behav. 124:107095. doi: 10.1016/j.addbeh.2021.107095, PMID: 34479068

[ref7] Attrill-Smith A. (2019). “The online self” in The Oxford handbook of cyberpsychology. eds. Attrill-Smith A. Fullwood C. Keep M. Kuss D. (Oxford: Oxford University Press), 17–34.

[ref8] Baker Z. G. Krieger H. LeRoy A. S. (2016). Fear of missing out: relationships with depression, mindfulness, and physical symptoms. Transl. Issues Psychol. Sci. 2, 275–282. doi: 10.1037/tps0000075

[ref9] Bányai F. Zsila Á. Király O. Maraz A. Elekes Z. Griffiths M. D.. (2017). Problematic social media use: results from a large-scale nationally representative adolescent sample. PLoS One 12:e0169839. doi: 10.1371/journal.pone.0169839, PMID: 28068404 PMC5222338

[ref10] Błachnio A. Przepiórka A. (2018). Facebook intrusion, fear of missing out, narcissism, and life satisfaction: a cross-sectional study. Psychiatry Res. 259, 514–519. doi: 10.1016/j.psychres.2017.11.012, PMID: 29154204

[ref11] Bollen K. A. Stine R. (1990). Direct and indirect effects: classical and bootstrap estimates of variability. Sociol. Methodol. 20, 115–140. doi: 10.2307/271084

[ref12] Brand M. Rumpf H. J. Demetrovics Z. Muller A. Stark R. King D. L.. (2020). Which conditions should be considered as disorders in the international classification of diseases (ICD-11) designation of "other specified disorders due to addictive behaviors"? J. Behav. Addict. 11, 150–159. doi: 10.1556/2006.2020.00035, PMID: 32634114 PMC9295220

[ref13] Brock R. D. (2017). “Gamification transformed: gamification should deliver the best parts of game experiences, not just experiences of game parts” in Transforming gaming and computer simulation technologies across industries. ed. Brock D. (Hershey, PA: IGI Global), 17–47.

[ref14] Brown N. (2021). “When seeing isn’t believing: Deepfakes and the war on truth” in Social media and society: An introduction to the mass media landscape. eds. Luttrell R. Wallace A. A. (Lanham, MD: Rowman & Littlefield), 13–32.

[ref15] Cao X. Masood A. Luqman A. Ali A. (2018). Excessive use of mobile social networking sites and poor academic performance: antecedents and consequences from stressor-strain-outcome perspective. Comput. Hum. Behav. 85, 163–174. doi: 10.1016/j.chb.2018.03.023

[ref16] Choi D. H. Noh G. Y. (2019). The influence of social media use on attitude toward suicide through psychological well-being, social isolation, and social support. Inf. Commun. Soc. 23, 1427–1443. doi: 10.1080/1369118X.2019.1574860

[ref17] Ciarrochi J. Hynes K. Crittenden N. (2005). Can men do better if they try harder: sex and motivational effects on emotional awareness. Cogn. Emot. 19, 133–141. doi: 10.1080/02699930441000102

[ref18] Cohen J. (1988). Statistical power analysis for the behavioral sciences. 2nd Edn. Mahwah, NJ: Lawrence Erlbaum Associates.

[ref19] Cohen J. F. Korevaar D. A. Altman D. G. Bruns D. E. Gatsonis C. A. Hooft L.. (2016). STARD 2015 guidelines for reporting diagnostic accuracy studies: explanation and elaboration. BMJ Open 6:e012799. doi: 10.1136/bmjopen-2016-012799, PMID: 28137831 PMC5128957

[ref20] Csikszentmihalyi M. (1990). Flow: The psychology of optimal experience. New York and London: HarperCollins.

[ref21] Csikszentmihalyi M. (2014). Applications of flow in human development and education: The collected works of Mihaly Csikszentmihalyi. Berlin: Springer.

[ref22] D’Arienzo M. C. Boursier V. Griffiths M. D. (2019). Addiction to social media and attachment styles: a systematic literature review. Int. J. Ment. Health Addict. 17, 1094–1118. doi: 10.1007/s11469-019-00082-5

[ref23] Dinçer E. Sayın M. Karadal H. (2022). “The fear of missing out (FoMO): the theoretical approach and measurement in organizations” in Handbook of research on digital violence and discrimination studies. ed. Özsungur F. (Hershey, PA: IGI Global), 631–652.

[ref24] Domoff S. E. Borgen A. L. Rye B. Barajas G. R. Avery K. (2022). “Problematic digital media use and addiction” in Handbook of adolescent digital media use and mental health. eds. Nesi J. Telzer E. H. Prinstein M. J. (Cambridge: Cambridge University Press), 300–316.

[ref25] Drouin M. Kaiser D. H. Miller D. A. (2012). Phantom vibrations among undergraduates: prevalence and associated psychological characteristics. Comput. Human Behav. 28, 1490–1496. doi: 10.1016/j.chb.2012.03.013

[ref26] El Abiddine F. Z. Aljaberi M. A. Gadelrab H. F. Lin C. Y. Muhammed A. (2022). Mediated effects of insomnia in the association between problematic social media use and subjective well-being among university students during COVID-19 pandemic. Sleep Epidemiol. 2:100030. doi: 10.1016/j.sleepe.2022.100030, PMID: 35992212 PMC9377837

[ref27] Fang J. Wang X. Wen Z. Zhou J. (2020). Fear of missing out and problematic social media use as mediators between emotional support from social media and phubbing behavior. Addict. Behav. 107:106430. doi: 10.1016/j.addbeh.2020.106430, PMID: 32289745

[ref28] Fox J. Vendemia M. A. (2016). Selective self-presentation and social comparison through photographs on social networking sites. Cyberpsychol. Behav. Soc. Netw. 19, 593–600. doi: 10.1089/cyber.2016.0248, PMID: 27732079

[ref29] Fullwood C. James B. M. Chen-Wilson C. J. (2016). Self-concept clarity and online self-presentation in adolescents. Cyberpsychol. Behav. Soc. Netw. 19, 716–720. doi: 10.1089/cyber.2015.0623, PMID: 27830930

[ref30] Fullwood C. Wesson C. Chen-Wilson J. Keep M. Asbury T. Wilsdon L. (2020). If the mask fits: psychological correlates with online self-presentation experimentation in adults. Cyberpsychol. Behav. Soc. Netw. 23, 737–742. doi: 10.1089/cyber.2020.0154, PMID: 32780589

[ref31] Geçikli F. (2020). “Digital addition” in New communication approaches in the digitalized world. eds. Erciş M. S. Başar E. E. (Cambridge: Cambridge Scholars Publishing), 1–12.

[ref32] Giordano A. L. (2022). A clinical guide to treating behavioral addictions. Berlin: Springer.

[ref33] Gori A. Topino E. Griffiths M. D. (2023). The associations between attachment, self-esteem, fear of missing out, daily time expenditure, and problematic social media use: a path analysis model. Addict. Behav. 141:107633. doi: 10.1016/j.addbeh.2023.107633 36753932

[ref34] Hayes A. F. (2022). Introduction to mediation, moderation, and conditional Process analysis: A regression-based approach. 3rd Edn. New York, NY: Guilford Press.

[ref35] Houghton S. Lawrence D. Hunter S. C. Rosenberg M. Zadow C. Wood L.. (2018). Reciprocal relationships between trajectories of depressive symptoms and screen media use during adolescence. J. Youth Adolesc. 47, 2453–2467. doi: 10.1007/s10964-018-0901-y, PMID: 30046970 PMC6208639

[ref36] Hunt M. G. Marx R. Lipson C. Young J. (2018). No more FOMO: limiting social media decreases loneliness and depression. J. Soc. Clin. Psychol. 37, 751–768. doi: 10.1521/jscp.2018.37.10.751

[ref37] Hussain Z. Griffiths M. D. (2021). The associations between problematic social networking site use and sleep quality, attention-deficit hyperactivity disorder, depression, anxiety and stress. Int. J. Ment. Health Addict. 19, 686–700. doi: 10.1007/s11469-019-00175-1

[ref38] Kaplan A. M. Haenlein M. (2010). Users of the world, unite! The challenges and opportunities of social media. Bus. Horiz. 53, 59–68. doi: 10.1016/j.bushor.2009.09.003

[ref39] Kim J. Y. Chung N. Ahn K. M. (2014). Why people use social networking services in Korea: the mediating role of self-disclosure on subjective well-being. Inf. Dev. 30, 276–287. doi: 10.1177/0266666913489894

[ref40] Krämer N. C. Winter S. (2008). The relationship of self-esteem, extraversion, self efficacy, and self-presentation within social networking sites. J. Media Psychol. 20, 106–116. doi: 10.1027/1864-1105.20.3.106

[ref41] Li L. Griffiths M. D. Mei S. Niu Z. (2020). Fear of missing out and smartphone addiction mediates the relationship between positive and negative affect and sleep quality among Chinese university students. Front. Psych. 11:877. doi: 10.3389/fpsyt.2020.00877, PMID: 33192635 PMC7481466

[ref42] Lin S. Longobardi C. Gastaldi F. G. M. Fabris M. A. (2024). Social media addiction and aggressive behaviors in early adolescents: the mediating role of nighttime social media use and sleep quality. J. Early Adolesc. 44, 41–58. doi: 10.1177/02724316231160142

[ref43] Lin L. Y. Sidani J. E. Shensa A. Radovic A. Miller E. Colditz J. B.. (2016). Association between social media use and depression among U.S. young adults. Depress. Anxiety 33, 323–331. doi: 10.1002/da.22466, PMID: 26783723 PMC4853817

[ref44] Malodia S. Otterbring T. Taheri B. Dhir A. (2024). How negative framing affects VR tourism adoption: exploring the role of travel anxiety during crisis events. J. Travel Res. doi: 10.1177/00472875241234387

[ref45] Manago A. M. Graham M. B. Greenfield P. M. Salimkhan G. (2008). Self-presentation and gender on Myspace. J. Appl. Dev. Psychol. 29, 446–458. doi: 10.1016/j.appdev.2008.07.001

[ref46] Marengo D. Angelo Fabris M. Longobardi C. Settanni M. (2022). Smartphone and social media use contributed to individual tendencies towards social media addiction in Italian adolescents during the COVID-19 pandemic. Addict. Behav. 126:107204. doi: 10.1016/j.addbeh.2021.107204, PMID: 34875508

[ref47] Marengo D. Settanni M. Fabris M. A. Longobardi C. (2021). Alone, together: fear of missing out mediates the link between peer exclusion in WhatsApp classmate groups and psychological adjustment in early-adolescent teens. J. Soc. Personal Relat. 38, 1371–1379. doi: 10.1177/0265407521991917

[ref48] Marriott T. C. Buchanan T. (2014). The true self online: personality correlates of preference for self-expression online, and observer ratings of personality online and offline. Comput. Hum. Behav. 32, 171–177. doi: 10.1016/j.chb.2013.11.014

[ref49] McFarlin D. B. Sweeney P. D. (1992). Distributive and procedural justice as predictors of satisfaction with personal and organizational outcomes. Acad. Manag. J. 35, 626–637. doi: 10.2307/256489

[ref50] McKinsey Health Institute. (2023). Gen Z mental health: The impact of tech and social media. Available online at: https://www.mckinsey.com/mhi/our-insights/gen-z-mental-health-the-impact-of-tech-and-social-media (Accessed November 19, 2024)

[ref51] Monacis L. Griffiths M. Limone P. Sinatra M. (2021). The risk of social media addiction between the ideal/false and true self: testing a path model through the tripartite person-centered perspective of authenticity. Telemat. Inform. 65:101709. doi: 10.1016/j.tele.2021.101709

[ref52] Murali V. George S. (2006). Lost online: an overview of internet addiction. Adv. Psychiatr. Treat. 13, 24–30. doi: 10.1192/apt.bp.106.002907

[ref53] Nadkarni A. Hofmann S. G. (2012). Why do people use Facebook? Pers. Individ. Dif. 52, 243–249. doi: 10.1016/j.paid.2011.11.007, PMID: 22544987 PMC3335399

[ref54] Neubaum G. Krämer N. C. (2015). My friends right next to me: a laboratory investigation on predictors and consequences of experiencing social closeness on social networking sites. Cyberpsychol. Behav. Soc. Netw. 18, 443–449. doi: 10.1089/cyber.2014.0613, PMID: 26252929

[ref55] Ng R. Indran N. (2023). Granfluencers on TikTok: factors linked to positive self-portrayals of older adults on social media. PLoS One 18:e0280281. doi: 10.1371/journal.pone.0280281 36749797 PMC9904471

[ref56] Ostic D. Qalati S. A. Barbosa B. Shah S. M. M. Galvan Vela E. Herzallah A. M.. (2021). Effects of social media use on psychological well-being: a mediated model. Front. Psychol. 12:678766. doi: 10.3389/fpsyg.2021.678766, PMID: 34234717 PMC8255677

[ref57] Pittman M. (2018). Happiness, loneliness, and social media: perceived intimacy mediates the emotional benefits of platform use. J. Soc. Media Soc. 7, 164–176.

[ref58] Podsakoff P. M. MacKenzie S. B. Lee J. Y. Podsakoff N. P. (2003). Common method biases in behavioral research: a critical review of the literature and recommended remedies. J. Appl. Psychol. 88, 879–903. doi: 10.1037/0021-9010.88.5.879, PMID: 14516251

[ref59] Primack B. A. Shensa A. Sidani J. E. Whaite E. O. Lin L. Y. Rosen D.. (2017). Social media use and perceived social isolation among young adults in the U.S. American. Am. J. Prev. Med. 53, 1–8. doi: 10.1016/j.amepre.2017.01.010, PMID: 28279545 PMC5722463

[ref60] Przybylski A. K. Murayama K. DeHaan C. R. Gladwell V. (2013a). Motivational, emotional, and behavioral correlates of fear of missing out. Comput. Hum. Behav. 29, 1841–1848. doi: 10.1016/j.chb.2013.02.014

[ref61] Przybylski A. K. Murayama K. DeHaan C. R. Gladwell V. (2013b). Fear of missing out scale (FoMOs). Available online at: 10.1037/t23568-000 (Accessed November 19, 2024)

[ref62] Radovic A. Gmelin T. Stein B. D. Miller E. (2017). Depressed adolescents' positive and negative use of social media. J. Adolesc. 55, 5–15. doi: 10.1016/j.adolescence.2016.12.002, PMID: 27997851 PMC5485251

[ref63] Rosen L. D. Carrier L. M. Pedroza J. A. Elias S. O’Brien K. M. Karina Kim J. L.. (2018). The role of executive functioning and technological anxiety (FOMO) in college course performance as mediated by technology usage and multitasking habits. Psicol. Educ. 24, 14–25. doi: 10.5093/psed2018a3, PMID: 33867798 PMC8048369

[ref64] Servidio R. Soraci P. Griffiths M. D. Boca S. Demetrovics Z. (2024). Fear of missing out and problematic social media use: a serial mediation model of social comparison and self-esteem. Addict. Behav. Rep. 19:100536. doi: 10.1016/j.abrep.2024.100536, PMID: 38495391 PMC10943642

[ref65] Shabahang R. Shim H. Aruguete M. S. Zsila A. (2023). Adolescent sadfishing on social media: anxiety, depression, attention seeking, and lack of perceived social support as potential contributors. BMC Psychol. 11:378. doi: 10.1186/s40359-023-01420-y, PMID: 37936212 PMC10631130

[ref66] Shewale R. (2024). Social media users 2024 (Global Data & Statistics). Available online at: https://www.demandsage.com/social-media-users/ (Accessed March 10, 2024)

[ref67] Skinner E. A. Belmont M. J. (1993). Motivation in the classroom: reciprocal effects of teacher behavior and student engagement across the school year. J. Educ. Psychol. 85, 571–581. doi: 10.1037/0022-0663.85.4.571

[ref68] Stead H. Bibby P. A. (2017). Personality, fear of missing out and problematic internet use and their relationship to subjective well-being. Comput. Hum. Behav. 76, 534–540. doi: 10.1016/j.chb.2017.08.016

[ref69] Strimbu N. O’Connell M. (2019). The relationship between self-concept and online self-presentation in adults. Cyberpsychol. Behav. Soc. Netw. 22, 804–807. doi: 10.1089/cyber.2019.0328 31794254

[ref70] Sun Y. L. Zhang Y. (2021). A review of theories and models applied in studies of social media addiction and implications for future research. Addict. Behav. 114:106699. doi: 10.1016/j.addbeh.2020.106699, PMID: 33268185

[ref71] Talan T. Doğan Y. Kalinkara Y. (2024). Effects of smartphone addiction, social media addiction and fear of missing out on university students’ phubbing: a structural equation model. Deviant Behav. 45, 1–14. doi: 10.1080/01639625.2023.2235870

[ref72] Wang X. L. (2021). Positive emotions’ role on social media addiction among Chinese young adults. Proceedings of the 2021 4th international conference on humanities education and social sciences (ICHESS 2021). Available online at: https://www.atlantis-press.com/proceedings/ichess-21/125966925

[ref73] Wang P. C. Xie X. C. Wang X. C. Wang X. Y. Zhao F. Q. Chu X. Y.. (2018). The need to belong and adolescent authentic self-presentation on SNSs: a moderated mediation model involving FoMO and perceived social support. Pers. Individ. Dif. 128, 133–138. doi: 10.1016/j.paid.2018.02.035

[ref74] Watson D. Clark L. A. Tellegen A. (1988). Development and validation of brief measures of positive and negative affect: the PANAS scales. J. Pers. Soc. Psychol. 54, 1063–1070. doi: 10.1037/0022-3514.54.6.1063, PMID: 3397865

[ref75] Wegmann E. Müller S. M. Turel O. Brand M. (2020). Interactions of impulsivity, general executive functions, and specific inhibitory control explain symptoms of social-networks-use disorder: an experimental study. Sci. Rep. 10:3866. doi: 10.1038/s41598-020-60819-4, PMID: 32123268 PMC7052241

[ref76] Winstone L. Mars B. Haworth C. M. A. Kidger J. (2021). Social media use and social connectedness among adolescents in the United Kingdom: a qualitative exploration of displacement and stimulation. BMC Public Health 21:1736. doi: 10.1186/s12889-021-11802-9, PMID: 34560872 PMC8464110

[ref77] Withington S. Punch A. (2019). There are costs from spending too much time on social media. Available online at: https://www.maxwell.syr.edu/research/lerner-center/population-health-research-brief-series/article/there-are-costs-from-spending-too-much-time-on-social-media#:~:text=Excessive%20social%20media%20use%20also,ability%20to%20develop%20meaningful%20relationships (Accessed November 19, 2024)

[ref78] Wolniewicz C. A. Tiamiyu M. F. Weeks J. W. Elhai J. D. (2018). Problematic smartphone use and relations with negative affect, fear of missing out, and fear of negative and positive evaluation. Psychiatry Res. 262, 618–623. doi: 10.1016/j.psychres.2017.09.058, PMID: 28982630

[ref79] World Health Organization (2022). International classification of diseases (11th rev, ICD-11). Geneva: World Health Organization.

[ref80] Young K. S. (1998). Caught in the net: How to recognise the signs of internet addiction and a winning strategy for recovery. Chichester, UK: John Wiley & Sons.

[ref81] Zarate D. Hobson B. A. March E. Griffiths M. D. Stavropoulos V. (2023). Psychometric properties of the Bergen social media addiction scale: an analysis using item response theory. Addict. Behav. Rep. 17:100473. doi: 10.1016/j.abrep.2022.100473, PMID: 36536822 PMC9758518

[ref82] Zhao S. Grasmuck S. Martin J. (2008). Identity construction on Facebook: digital empowerment in anchored relationships. Comput. Hum. Behav. 24, 1816–1836. doi: 10.1016/j.chb.2008.02.012

[ref83] Zimmer-Gembeck M. J. (2016). Peer rejection, victimization, and relational self-system processes in adolescence: toward a transactional model of stress, coping, and developing sensitivities. Child Dev. Perspect. 10, 122–127. doi: 10.1111/cdep.12174

